# Noise exposure among staff in intensive care units and the effects of unit-based noise management: a monocentric prospective longitudinal study

**DOI:** 10.1186/s12912-023-01611-3

**Published:** 2023-12-06

**Authors:** Christoph Armbruster, Stefan Walzer, Sandra Witek, Sven Ziegler, Erik Farin-Glattacker

**Affiliations:** 1 Institute of Medical Biometry and Statistics (IMBI), Section of Health Care Research and Rehabilitation Research (SEVERA), Medical Center – University of Freiburg, Faculty of Medicine, University of Freiburg, Freiburg, 79106 Germany; 2 Faculty of Health, Safety and Society, Care and Technology Lab, Furtwangen University, 78120 Furtwangen, Germany; 3 Center of Implementing Nursing Care Innovations Freiburg, Medical Center – University of Freiburg, 79106 Freiburg, Germany

**Keywords:** Intensive care units, Noise, Health, Health personnel, Intervention

## Abstract

**Background:**

Intensive care units (ICUs) are often too noisy, exceeding 70–80 dBA, which can have negative effects on staff. The corresponding recommendation of the World Health Organization (average sound pressure level below 35 dBA) is often not achieved. To date there is a lack of intervention studies examining the extent to which unit-based noise management in ICUs contributes to a reduction in noise exposure for the staff. The study therefore aims to provide answers to 1) how unit-based noise management sustainably reduces the subjective noise exposure among staff, and 2) how this intervention affects other noise-related topics.

**Methods:**

We performed a monocentric prospective longitudinal study with three measurement points in a German university hospital in three ICUs. We collected data from different healthcare professionals and other professional groups between October 2021 and August 2022 using an online questionnaire. Data were analyzed using descriptive and inference statistics.

**Results:**

A total of *n* = 179 participants took part in the surveys. The majority of participants were nurses or pediatric nurses. Most participants worked more than 75% full-time equivalent. Staff on the three ICUs reported high levels of noise exposure. No significant changes in noise exposure over time were observed. Participants were already aware of the topic and believed that a behavior change could positively influence the noise environment.

**Conclusions:**

This study provides an initial insight into how a unit-based noise management could contribute to a reduction in the subjective noise exposure among staff in ICUs. The results of this study highlight the importance of this topic. Future studies should aim to research aspects of adherence and their facilitators or barriers, which promote the sustained implementation of noise-reducing measures by staff.

**Trial registration:**

German Clinical Trials Register (DRKS): DRKS00025835; Date of registration: 12.08.2021.

**Supplementary Information:**

The online version contains supplementary material available at 10.1186/s12912-023-01611-3.

## Background

Intensive care units (ICUs) are designed to provide care for critically ill patients who require special attention and treatment [1]. They are equipped with a high number of medical devices to inform staff (e.g. nurses) about the health status of patients [2]. Whilst this ensures an improvement in medical and nursing care, it also contributes to significant noise emission [3]. Noise can have various causes and is not always predictable [4]. A categorization can be made into two areas: 1) Device-generated noise, such as mechanical noise [5] or alarms [6, 7] and 2) noise generated by people, such as conversations among staff and relatives [8, 9] or medical and nursing activities [10, 11].

Noise can be considered as sound, which transports energy as a mechanical wave [12]. Sound itself is measured in the form of several technical quantities, such as the sound pressure level (unit dB) [13]. However, the perception of sound also depends to the timbre, the tonality and impulsiveness [14]. In addition personal characteristics (e.g. cultural background, human hearing) can influence the perception of sound. Moreover, perceptions may also differ in terms of physiological and psychological factors (e.g. health status, self-efficacy) [15, 16]. In this context, the sound pressure level is filtered during measurement to take these characteristics into account (unit dBA: A-weighted decibel scale) [16, 17]. According to Berglund et al. [18], the average sound pressure level (LAeq) in ICUs should not exceed 35 dBA. A more precise distinction is made in the recommendations of the "German Interdisciplinary Association of Intensive Care and Emergency Medicine (DIVI)", whereby the limits are classified by daytime (i.e. maximum 45 dB during the day, 40 dB in the evening, 20 dB at night) [19]. However, international studies have shown that sound pressure levels in ICUs have increased over the last 50 years and recommendations are being significantly exceeded [20–23].

The sensitive character of an ICU has already led to increased research into noise [2, 24–26]. Different studies have shown that noise induces stress reactions [27], which are also predictors of various symptoms and diseases (e.g. fatigue, exhaustion [28], anxiety [29], burnout, or depression [28, 30, 31]. Furthermore, noise generated by acoustic (false) alarms can affect the behavior of medical staff in terms of setting wider alarm limits or reducing their volume [7]. In addition, (false) alarms may also contribute to desensitization (alarm fatigue) [32, 33] which can affect patient safety (e.g. no reaction in case of a "real" alarm). Moreover, such (false) alarms can lead to annoying interruptions [3, 34] as well as errors in medical activities (e.g. preparing medications) [27, 35]. According to Sengpiel [17], this is already possible at a sound pressure level of 40 dBA.

Noise, however, is not the only challenge medical staff face in ICU [36]. A high workload [37], which might contribute to job dissatisfaction [38], and massive staff shortages [39] are but a few aspects to note. Since the Covid-19 pandemic, these challenges have intensified even further [40]. Thus, it is important to implement measures that reduce the burden on staff in ICUs. In this regard, one strategy can be the sustainable reduction of noise.

In a review, Konkani and Oakley [2] describe several approaches to noise reduction in ICUs. Besides measures to change the behavior of staff (e.g. through education or noise visualization), other options include quiet times, station remodeling, or the volume adjustment of technical devices (e.g. television, telephone). However, considering the individual situation of an ICU, the authors conclude that a standardized approach to noise reduction is not realistic [2].

Up to now, research on noise has mainly focused on objective sound measurements [1, 21, 41, 42] or on the patient's perspective [43, 44]. Staff and their subjective noise exposure, has mostly been recorded in cross-sectional surveys [10, 26, 28, 29]. To date there is a lack of intervention studies examining the extent to which unit-based noise management in ICUs contributes to a reduction in noise exposure among staff. The study therefore aims to provide an answer to the following research questions: 1) Can unit-based noise management sustainably reduce the subjective noise exposure of staff in ICUs? 2) How does unit-based noise management affect other noise-related topics (e.g. knowledge and awareness or thematization to noise)?

## Methods

We report this study using the Strengthening the Reporting of Observational Studies in Epidemiology (STROBE-) Statement [45]. The study was registered in the German Clinical Trials Register (DRKS00025835).

### Studydesign and setting

**Fig. 1:**
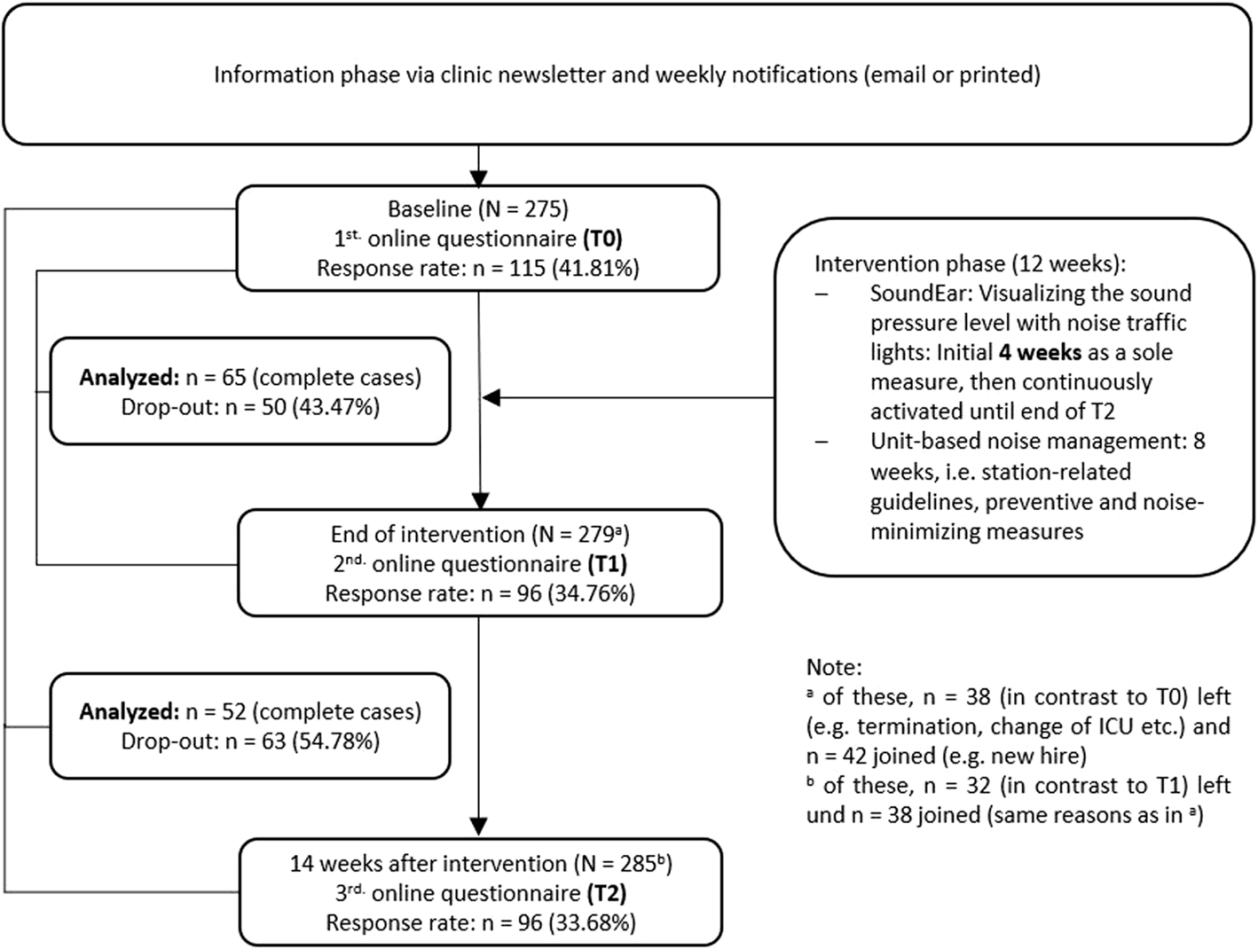
Process of data collection and intervention

We conducted a monocentric prospective longitudinal study with three measurement points (MPs; labeled as T0, T1 and T2) at the University Medical Center Freiburg (maximum care) in three selected ICUs (i.e. anesthesiological, neonatological, and neurological). We chose this study design to be able to assess changes in the data collected over time and thus determine the effects of the intervention [46]. The time span between T0-T1 was 12 weeks and between T1-T2 was 14 weeks (Fig. 1). Data collection took place between October 2021 and August 2022.

### Participants

Participants were staff on three ICUs who were informed in advance about the study project and objectives via the clinic newsletter and weekly notifications (via email or paper-based). Since a full coverage survey was aimed at for each MP, no sample size calculation was performed. To counteract drop-out in longitudinal studies [47], a fundraising campaign was additionally initiated at the last MP. For each completed questionnaire, 3 euros could be donated to a social organization selected in advance by the respective ICU. Table 1 shows the inclusion and exclusion criteria of the study participants.

**Table 1:** Inclusion and exclusion criteria

**Inclusion criteria:**
1. ≥ 18 years
2. Staff in one of the three ICUs, i.e
a. Nurses, pediatric nurses or nursing assistants
b. Staff from other nursing support professions, e.g. service assistants, hotel staff, secretaries
c. Physicians
d. Physio-, occupational- or speech therapy staff
**Exclusion criteria:**
1. Trainees in nursing or therapeutic professions
2. Students of human medicine
3. "Reserve pool" staff

### Intervention

The intervention contained three strategies (i.e. noise traffic lights, implementation of guidelines, further unit-based measures). Table 2 presents an overview of different measures within these strategies. The study process can be seen in Fig. 1.

**Table 2:** Measures of the intervention

**Noise traffic lights**	Visualizing the thresholds of sound pressure level with noise traffic lights (company: SoundEar A/S; model: SoundEar® 3 310 [48]). For positioning, the noisiest locations were identified in advance (i.e. before the actual intervention) by working groups in the respective ICUs^a^. In each ICU, we installed a total of 3 noise traffic lights [48]. Depending on ICU and localization, the limits of the noise traffic lights were set between 59–67 dBA^b^ for the yellow light and between 62–70 dBA for the red light
**Implementation of guidelines**	The guidelines included immediate and medium-term measures, as well as measures with low and high organizational effort [18]. In addition, the guidelines considered the individual circumstances of the stations (e.g. type of staff, internal processes). For instance, the following measures were implemented: individual alarm management; adjustment of work processes, such as the restructuring of rounds; telephone calls and conversations outside the patient rooms or in a quiet manner; collegial advice during loud conversations; quiet times
**Further unit-based measures**	These included preventive and noise-reducing measures: education through one-minute-wonders and informative material, such as posters regarding noise and its consequences; transparency through regular reporting of the interim results of the sound pressure level measurements; checking the correct adjustment of noise-generating technical devices; using stickers and postcards to inform external staff, visitors and relatives about the noise issue

^a^ Intensiv care unit(s)

^b^ A-weighted decibel scale

### Data collection

For the online surveys, we used a slightly modified version of the questionnaire from the study by Schmidt et al. [10] with a total of 19 items. The questionnaire included items on the following topics: noise-related strain, knowledge and awareness, thematization, subjective noise-sensitivity, attitude to alternative alarm systems, disturbance from20 different noise sources. In addition to the items from Schmidt et al. [10], we included the item "Do you think it is very important to thematize noise on ICU?" in the topic of thematization to ascertain the significance of noise management according to the staff's perspective. Questions could be answered via a 4-point Likert scale (i.e. 1 = "no", 2 = "rather no", 3 = "rather yes", 4 = "yes"). Perceived disturbance from the noise sources was measured from 1 = "not at all disturbing" to 4 = "very disturbing", supplemented with the answer category of "don't know". Furthermore, we added an item on general noise exposure (i.e. noise exposure outside of work) [49] and items on sociodemographic characteristics. After this, we conducted a pretest to check content validity through expert rating. For this, 10 researchers (partly directly involved in the project and with additional professional experience in an ICU or at least having knowledge of the contents and objectives of the project) answered closed-ended and open-ended items (e.g. content, order, clarity or processing time). The questionnaire was designed in REDCap [50] after a final revision that included linguistic adaptation of the topic introductions and the addition of another noise source (cleaning work) to the total of 21.

At each MP, a personalized survey link was distributed via the professional email addresses of the staff, which we obtained through the intranet or gatekeepers (i.e. unit leaders). To ensure that participants did not take part in a survey multiple times, this link could only be used once per MP. Staff provided their informed consent online prior to answering the surveys. For each MP, we reminded the staff to participate twice by email (2 weeks apart) and we also approached staff directly in the ICU through personal contact.

### Statistical analysis

Data were analyzed using descriptive and inference statistics. Before analysis, we performed plausibility checks and prepared the data according to the coding manual. Regarding the first research question, we proceeded as follows: Since the questionnaire in the original publication [10] was not checked for unidimensionality, we tested the relevant items regarding noise-related strain using confirmatory factor analysis (CFA). To capture the change in noise-related strain over time, we performed pre-post comparisons (i.e. T0-T1 resp. T0-T2) using t-test for paired samples (complete case analysis (CCA)). Due to a high correlation in repeated measurements, we calculated the associated corrected effect sizes [51]. To counteract biased estimation (i.e. only CCA) [52], noise-related strain in ICUs was additionally considered using a linear mixed model (LMM) (i.e. considering all the observations, regardless of how often they participated in the surveys and with adjustment for the variable "general noise exposure"). For this, we first adapted the very unequal scale value of the variable "general noise exposure" (range: 0–100) to the dependent variable (range: 1–4), dividing the values of the variable "general noise exposure" by 10 to simplify the interpretation of the regression coefficients. In addition, prior suitability testing was carried out (i.e. consideration of the intraclass correlation coefficient (ICC)). Concerning the second research question, we conducted all the analyses on an item level (due to missing unidimensionality in items with similar content or underidentified models within the CFAs). Pre-post comparisons (i.e. T0-T1 resp. T0-T2) were analyzed using the Wilcoxon rank-sum test. To determine the specific direction of change for equal median and statistical significance, the respective distributions (pre-post) were compared with each other. Due to an exploratory approach, a restrictive *p*-value adjustment was not applied [53]. For all analyses, statistical significance was set at *p* < 0.05. Missing values were considered descriptively and separately by the respective MP after exclusion of the variables for which no missing values were possible (e.g. ID). For all the analyses we used R (Version 4.0.2) [54] and R Studio (Version 2022.07.1) [55] with the packages psych, lavaan, lme4, nlme, rstatix.

## Results

### Sample characteristics

A total of *n* = 179 participants (who completed at least one survey) took part in this study. At T0, the response rate was 41.81%, at T1 = 34.76%, and at T2 = 33.68% (Fig. 1). For each MP, the age group from 30 to under 40 was the most represented. Approximately two thirds of the participants were female. The majority of participants were nurses or pediatric nurses (i.e. > 50% at each MP). In terms of all professional groups and MPs, the largest proportion was those with more than 15 years of work experience (i.e. approximately 30%). Most participants worked more than 75% full-time equivalent. Exact details of the sample characteristics per MP can be seen in Table 3.

**Table 3:** Sample characteristics per MP

	**T0**	**T1**	**T2**
**Sample size**			
n	115	96	96
**Age**			
until below 30	25 (22.9%)	21 (22.8%)	22 (23.7%)
30 to under 40	32 (29.4%)	31 (33.7%)	32 (34.4%)
40 to under 50	26 (23.9%)	22 (23.9%)	18 (19.4%)
50 and older	26 (23.9%)	17 (18.5%)	21 (22.6%)
no specification/missing	6 (5.2%)	5 (5.2%)	3 (3.1%)
**Gender**			
female	74 (66.7%)	62 (67.4%)	58 (62.4%)
male	36 (32.4%)	26 (28.3%)	31 (33.3%)
no specification/missing	5 (4.3%)	8 (8.3%)	7 (7.3%)
**ICU**			
anesthesiological	30 (27.0%)	19 (20.9%)	24 (26.1%)
neonatological	45 (40.5%)	45 (49.5%)	38 (41.3%)
neurological	35 (31.5%)	27 (29.7%)	29 (31.5%)
no specification/missing	5 (4.3%)	5 (5.2%)	5 (5.2%)
**Professional group**			
nurses or pediatric nurses	57 (51.8%)	48 (54.5%)	51 (54.8%)
physicians	22 (20.0%)	17 (19.3%)	19 (20.4%)
other^a^	29 (26.4%)	21 (23.9%)	22 (23.7%)
no specification/missing	7 (6.1%)	10 (10.4%)	4 (4.2%)
**Work experience** (in years)			
until below 2	15 (13.6%)	14 (15.6%)	15 (16.3%)
2 to under 5	20 (18.2%)	16 (17.8%)	14 (15.2%)
5 to under 10	26 (23.6%)	20 (22.2%)	21 (22.8%)
10 to under 15	16 (14.5%)	12 (13.3%)	12 (13.0%)
15 and more	32 (29.1%)	27 (30.0%)	29 (31.5%)
no specification/missing	6 (5.2%)	7 (7.3%)	5 (5.2%)
**Work proportion** (in %)			
until below 75	39 (35.5%)	24 (26.4%)	28 (30.8%)
75 to 100	69 (62.7%)	65 (71.4%)	62 (68.1%)
no specification/missing	7 (6.1%)	7 (7.3%)	6 (6.2%)

^a^ e.g. physio-, occupational- or speech therapy, nursing assistants, service assistants, staff from hotels, secretaries

### Missing values

The surveys contained only a small proportion of missing values per MP. Per variable surveyed, average missing values were at T0: 4.23% (SD = 1.67%), at T1: 3.36% (SD = 1.55%), and at T2: 4.00% (SD = 1.95%). At the individual level, there was an average of < 2.00% missing values (i.e. T0 = 1.65% (SD = 6.70%), T1 = 1.73% (SD = 7.40%), and T2 = 1.96% (SD = 8.13%). The questionnaire was fully completed (i.e. no missing values) at T0 by 63.47%, at T1 by 60.41%, and at T2 by 64.58%. The drop-out between T0-T1 was *n* = 50 (43.47%) and between T0-T2 the drop-out rate was *n* = 63 (54.78%). Thus, the number of complete cases (CC) between T0-T1 was *n* = 65 (56.53%), between T0-T2 *n* = 52 (45.22%). A total of *n* = 41 (35.65%; referring to T0) participated in all the surveys. No significant differences were found between drop-outs and CC with respect to sociodemographic variables.

### Noise-related strain

Using the baseline data (T0), 5 items were tested for unidimensionality and summarized to the scale of "noise-related strain":

1. Do you sometimes feel sometimes disturbed by the ambient noise while working?
2. Do you think the ambient noises in the ICU affect your working performance?
3. Do you sometimes feel irritated by the ambient noises in the ICU?
4. Do you think the ambient noises in the ICU affect your well-being?
5. Does the noise in the ICU fatigue you?

The CFA yielded an acceptable model fit (*p*-value of Chi-Square-difference test = 0.181; CFI = 0.94, TLI = 0.93, SRMR = 0.08). Cronbach`s alpha was 0.86, 95% CI [0.82, 0.90], indicating a very good inter-item correlation [56]. The scale ranges from 1 = "no noise-related strain" to 4 = "high noise-related strain".

Descriptively, noise-related strain among the staff in the ICU was rather high (i.e. approximately 3 in a possible range of 1–4). With respect to the CC, the means between T0-T1 were 3.13 (SD = 0.64) and 3.08 (SD = 0.73), whereas between T0-T2 they were 2.99 (SD = 0.67) and 2.97 (SD = 0.76), respectively. The t-test for paired samples (CCA) showed no significant differences between T0-T1 as well as T0-T2 (t = 0.784; df = 61; *p* = 0.436 and t = 0.337; df = 49; *p* = 0.738, respectively). Intervention effects regarding a reduction in noise-related strain were not identified (Cohen`s d = 0.07, 95% CI [-0.28, 0.42] and Cohen`s d = 0.03, 95% CI [-0.36, 0.42], respectively).

To consider all the participants who took part in the survey for at least one MP, we additionally analyzed the data using an LMM. For model specification, we initially estimated the ICC of the ICU-clusters and the clusters for repeated measurements, on a personal level based on null models. The ICC of the ICU-clusters was zero, resulting in exclusion from the model. In contrast, the ICC on the personal level was 0.64, which led to inclusion (level 2). Table 4 shows the results of the LMM. The intercept represents the noise-related strain of ICU staff at T0 (2.932). Controlled for general noise exposure a reduction in noise-related strain was observed over time, but not significantly. For each positive unit-change in general noise exposure, there was a significant increase in noise-related strain by 0.042 units (controlled for time).

**Table 4:** LMM of noise-related strain in the ICU

	**Noise-related strain (all observations)**	
**Fixed effects**		
Intercept	**2.932**	(0.088)
Time [T1]	-0.091	(0.056)
Time [T2]	-0.075	(0.060)
General noise exposure	**0.042**	(0.013)
**Random effects**		
Intercept	0.337	
Residual	0.117	

Note: Unstandardized regression coefficients with standard error in parentheses; values in bold are statistically significant with *p* < 0.05; general noise exposure: noise exposure outside the work

### Other noise-related topics

As regards the CC (T0-T1 resp. T0-T2) and their baseline data (T0), the majority of the staff assume that the guideline of the WHO (35–40 dBA) cannot (54.2% resp. 55.3%) or rather cannot (39.0% resp. 46.0%) be complied with. Changing one's own behavior can contribute to noise reduction (rather yes: 45.2% resp. 42.0%; yes: 27.4% resp. 26.0%). The thematization of noise was felt to be very important (rather yes: 17.7% resp. 26.0%; yes: 82.3% resp. 74.0%). Noise is more likely to be thematized among colleagues (rather yes: 14.5% resp. 24.0%; yes = 48.4% resp. 36.0%) rather than with superiors (rather yes: 11.3% resp. 10.2%; yes = 29.0% resp. 24.5%) or in private life (rather yes: 18.8% resp. 22.0%; yes: 21.3% resp. yes = 14.0%). In addition, staff indicated that they increasingly seek rest after a shift (rather yes: 38.7% resp. 42.0%; yes = 32.3% resp. 30.0%). In terms of alternative alarm systems, staff considered vibrating solutions to be more helpful than visible solutions. The relative frequencies for all the items can be seen in Additional file 1. Between T0-T1, significant changes were recorded for 3 items in the area of "thematization", and between T0-T2 for one item in the area of "knowledge and awareness". Table 5 provides an overview of the changes in the pre-post comparison (T0-T1 resp. T0-T2) and the corresponding effect sizes.

**Table 5:** Pre-post comparisons regarding to other noise-related topics

	**T0-T1**					**T0-T2**				
	n	Mdn^a^ T0	Mdn T1	p	r^b^ [CI]^c^	n	Mdn T0	Mdn T2	p	r [CI]
**Knowledge and awareness**										
The World Health Organization recommends that the noise level in the hospital should not exceed 35–40 dBA (unit for noise level). The example "room ventilator" = 35 dBA should give you an orientation. Do you think this guideline is implemented most of the time?	59	1	2	0.132	0.199 [0.030, 0.430]	47	1	2	0.008^d^	0.391 [0.110, 0.610]
Do you experience the sounds on the ICU as too loud?	62	3	3	0.868	0.007 [0.002, 0.290]	50	3	3	1.000	0.021 [0.005, 0.310]
Do you think it would be possible to reduce noise levels on the ICU, by changing your own behavior?	62	3	3	0.722	0.003 [0.005, 0.270]	50	3	3	0.988	0.017 [0.007, 0.330]
**Thematization**										
Do you think it is very important to thematize noise on ICU?	62	4	4	0.042^d^	0.256 [0.040, 0.470]	50	4	4	0.594	0.082 [0.000, 0.330]
Do you talk with your colleagues about the ICU ambient noise?	62	3	3	0.395	0.097 [0.004, 0.350]	50	3	3	0.882	0.029 [0.006, 0.320]
Is the ambient noise on the ICU an issue in discussions with your superiors?	62	2	2	1.000	0.023 [0.002, 0.280]	49	2	2	0.916	0.053 [0.007, 0.320]
Do you talk with your family/friends about the noise on the ICU?	61	2	2	0.005^d^	0.344 [0.110, 0.540]	50	2	2	0.858	0.028 [0.003, 0.330]
Are you addressed by patients or their dependents about the noise levels on the ICU?	61	2	2	0.047^d^	0.299 [0.070, 0.510]	50	2	2	0.184	0.194 [0.010, 0.470]
**Subjective noise-sensitivity**										
Are you seeking increased for calm after a working shift on the ICU?	62	3	3	0.614	0.037 [0.006, 0.290]	50	3	3	0.771	0.052 [0.004, 0.330]
Do you feel being lesser in the mood for listening to music after working?	61	2	2	0.283	0.170 [0.010, 0.410]	49	2	2	0.819	0.049 [0.005, 0.330]
Compared to other people who don´t work on the ICU: Do you react more sensitive to sounds after having had a shift on the ICU?	56	3	3	0.260	0.104 [0.006, 0.360]	47	3	3	0.276	0.135 [0.005, 0.390]
**Attitude towards alternative alarm systems**										
Do you think, vibrating alarm signals (e.g. by a smart watch) could replace auditive ones (e.g. a call system)?	61	3	3	0.771	0.043 [0.003, 0.310]	50	3	3	0.776	0.024 [0.003, 0.310]
Do you think, visible alarm signals (e.g. by a smart watch) could replace auditive ones (e.g. a call system)?	61	3	2	0.154	0.155 [0.008, 0.400]	49	3	3	0.617	0.064 [0.005, 0.350]

^a^ median

^b^ 0.1–0.3 = small effect, 0.3–0.5 = medium effect, >  = 0.5 = large effect

^c^ 95% confidence interval

^d^ positive change

### Perceived disturbance to individual noise sources

Regarding the perceived disturbance of individual noise sources, we performed a subdivision into "technical devices" and "clinical activities or actions". Again, the following results refer to the CC (i.e. T0-T1 resp. T0-T2) and their baseline statements (T0). In terms of technical devices, staff most often rated surveillance monitors (alarms) as rather disturbing (30.6% resp. 41.7%) or very disturbing (54.8% resp. 45.8%). Furthermore, mechanical ventilators (rather disturbing: 44.3% resp. 40.8%; very disturbing: 24.6% resp. 24.5%), as well as perfusors (rather disturbing: 48.4% resp. 48.0%; very disturbing: 21.0% resp. 18.0%) or telephones (rather disturbing: 35.5% resp. 30.0%; very disturbing: 43.5% resp. 40.0%) were perceived as disruptive noise sources. In the area of clinical activities or actions, the results show that staff rated the private conversations of colleagues as disturbing (rather disturbing: 40.3% resp. 32.0%; very disturbing: 33.9% resp. 28.0%). In addition, the use of the brake on the bed (rather disturbing: 24.6% resp. 18.0%; very disturbing: 37.7% resp. 38.0%), visits (rather disturbing: 33.9% resp. 34.0%; very disturbing: 22.6% resp. 22.0%), and the opening of cartons or packages (rather disturbing: 19.0% resp. 20.8%; very disturbing: 39.7% resp. 37.5%) were frequently perceived as interfering noise sources. Additional file 2 shows the relative frequencies for all noise sources. Between T0-T1, 2 noise sources showed significant changes in the area of "clinical activities or actions" (i.e. using the brake on the bed, shoes (e.g. squeaking)), however the first one showed negative changes. A significant change (negative) between T0-T2 was found in the area of "technical devices" for one noise source (i.e. compressed air). Table 6 shows all the changes in the pre-post comparison (i.e. T0-T1 resp. T0-T2) with the corresponding effect sizes. When investigating other noise sources (free text question), the nutrition pump was mentioned most frequently (based on all cases per MP) (i.e. T0: *n* = 7; T1: *n* = 8; T2: *n* = 8).

**Table 6:** Pre-post comparisons on the perceived disturbance of individual noise sources

	**T0-T1**					**T0-T2**				
	n	Mdn^a^ T0	Mdn T1	p	r^b^ [CI]^c^	n	Mdn T0	Mdn T2	p	r [CI]
**Technical devices**										
Mechanical ventilators	58	3	3	0.603	0.040 [0.003, 0.290]	48	3	3	0.425	0.102 [0.000, 0.370]
Surveillance monitors (alarms)	62	4	3	1.000	0.038 [0.005, 0.310]	48	3	3	0.745	0.087 [0.004, 0.380]
Dialysis machine	39	3	3	0.212	0.117 [0.008, 0.420]	35	3	3	0.086	0.277 [0.030, 0.570]
Perfusors	58	3	3	0.400	0.087 [0.006, 0.320]	48	3	3	0.240	0.160 [0.009, 0.430]
ECMO^f^	20	2	2	0.530	0.158 [0.000, 0.520]	19	2	2	1.000	0.000 [N/A^g^]
Suction pump	50	2	3	0.150	0.184 [0.010, 0.450]	41	2	3	0.417	0.065 [0.008, 0.360]
Visitor bell	58	3	3	0.964	0.052 [0.005, 0.320]	48	3	3	0.822	0.027 [0.005, 0.340]
Telephones	59	3	3	0.697	0.012 [0.006, 0.310]	50	3	3	0.244	0.173 [0.007, 0.420]
Beeper	52	2	2	0.736	0.035 [0.000, 0.310]	41	2	3	0.065	0.359 [0.060, 0.620]
Heated blanket	34	2	2	0.884	0.044 [0.000, 0.400]	27	2	3	0.358	0.232 [0.010, 0.560]
Compressed air	33	2	2	0.287	0.124 [0.010, 0.440]	35	2	2	0.018^e^	0.403 [0.110, 0.670]
Thoracic drainage	39	2	2	0.674	0.049 [0.000, 0.370]	33	2	2	0.830	0.011 [0.000, 0.380]
Transport monitor / ventilator	51	2	2	0.985	0.021 [0.006, 0.310]	43	2	2	0.414	0.155 [0.008, 0.440]
**Clinical activities or actions**										
Using the brake on the bed	50	3	4	0.009^e^	0.353 [0.110, 0.560]	40	3	4	0.082	0.310 [0.050, 0.570]
Opening cartons or packages	56	3	3	0.327	0.054 [0.004, 0.310]	47	3	3	0.674	0.097 [0.006, 0.360]
Opening or closing doors/drawers	62	2	3	0.386	0.115 [0.007, 0.360]	49	2	2	0.866	0.027 [0.002, 0.340]
Visits	62	3	3	0.740	0.060 [0.002, 0.300]	50	3	3	0.386	0.099 [0.004, 0.370]
Private conversations from colleagues	60	3	3	0.597	0.059 [0.002, 0.290]	48	3	3	1.000	0.022 [0.000, 0.310]
Cleaning work	62	2	2	0.622	0.069 [0.006, 0.310]	49	2	2	0.106	0.211 [0.010, 0.470]
Shoes (e.g. squeaking)	58	2	2	0.024^d^	0.279 [0.060, 0.500]	48	2	2	0.217	0.211 [0.007, 0.480]
Conversation of visitors	57	2	2	0.528	0.068 [0.004, 0.330]	47	2	2	0.829	0.035 [0.000, 0.340]

^a^ median

^b^ 0.1–0.3 = small effect, 0.3–0.5 = medium effect, >  = 0.5 = large effect

^c^ 95% confidence interval

^d^ positive change

^e^ negative change

^f^ extracorporeal membrane oxygenation

^g^ *N/A* not applicable

## Discussion

To the best of our knowledge, there is an evidence gap on how unit-based noise management can sustainably reduce the subjective noise exposure and how this intervention affects other noise-related topics among staff in ICUs. We therefore conducted this study to gain deeper insights into this field of research. Overall, we were unable to identify significant changes in noise exposure after implementation of noise management. Furthermore, comparisons with other studies are difficult because these works mainly used cross-sectional designs or did not focus on staff behavior and experiences.

### Noise-related strain

In our study, the staff in the three ICUs reported high levels of noise exposure. This finding is consistent with the study by Schmidt et al. [10], which surveyed 348 healthcare professionals in ICUs in the German-speaking part of Switzerland. A recent study in a similar setting (*n* = 350) also confirmed our results, with more than two-thirds of the participating staff perceiving the ICU as being too loud [26]. These results reinforce the importance of noise management in ICUs. As regards the effect of the implemented noise management, however, we were unable to observe a significant reduction in the perceived noise-related strain over time. This could be due to the constant high sound pressure levels, which will be published in another part of the overall study (Witek et al. forthcoming). According to Kebapci and Güner [57] one reason for the persistently high noise level could be that measures to reduce noise only have a short-term effect if the entire staff on the ICU do not consistently respect them. This seems to be a challenge since the majority of the surveyed nurses stated that they had become insensitive to the constant noise exposure [57]. However, it should be noted, that behavioral change strategies are considered to be the most effective and cost-effective approach for long-term noise reduction [2, 42]. Such strategies include educational programs and campaigns to inform staff about the harmful effects of noise exposure and ways to mitigate it or to establish a culture of accountability for noise reduction among staff, with regular reminders and discussions about the importance of a quiet environment for patients and staff [2, 10, 58, 59].

### Other noise-related topics

With regard to "knowledge and awareness", this study revealed that the surveyed individuals were already aware of the issue of noise prior to the implementation of noise management measures. In contrast to this, Johannson et al. [60] showed that staff in ICU have a lack of theoretical knowledge about noise and its negative consequences. The respondents in our study believed that changes in their own behavior (e.g. addressing alarms efficiently to prevent unnecessary noise) could contribute to noise reduction in their own ICUs. These findings are in line with those of Schmidt et al. [10] and Ruettgers et al. [26], who also found that staff were aware of the issue of noise exposure and believed that changes in their own behavior could positively influence the noise environment. Nevertheless, Ruettgers et al. [26] showed that staff perceived changes in technical equipment and adjustments to alarms as a more straightforward solution in noise management. This is also confirmed by another study in which technical modifications led to a significantly lower sound pressure level [61]. However, this could be difficult because staff assume that superiors are not open to such modification [27]. As far as changes over time are concerned, we found a positive significant difference between T0 and T2 for only one item (i.e. "The World Health Organization recommends […]. Do you think this guideline is implemented most of the time?"). One explanation for this may be the initial high level of knowledge and awareness among the staff about this topic. After all, the intervention also aims to increase knowledge and awareness of noise, which could be considered as achieved, as indicated by the unchanged or positively altered median values of the corresponding items between T0-T1 and T0-T2 (see Table 5). In terms of "thematization", it is apparent that staff felt it was important to discuss the topic of "noise". This also indicates that the sense of disturbance is high and thus confirms our descriptive results of noise-related strain. As in the study by Ryherd et al. [27], the exchange is most likely to take place among colleagues. It is known that superiors and subordinates often have different perceptions of responsibilities, which can lead to low quality communication between them [62]. Overall, we found a positive change in three items (T0-T1), which could indicate a decrease in the noise levels in the ICUs. In the context of "subjective noise-sensitivity", it appears that participants are more likely to seek quiet and are more sensitive to noise after a shift in the ICU. These results are comparable to those of Schmidt et al. [10] and support the findings of high noise-related strain, regardless of the MPs.

### Individual noise sources

As expected, different noise sources (i.e. in the area of technical devices or clinical activities and actions) are perceived as disturbing in the ICU. Previous studies [10, 59, 63] showed that mechanical ventilators, monitors and their alarms, telephones conversations or visits are highly disruptive to ICU staff which is consistent with our results. Unfortunately, we hardly found any significant change over time in the noise sources we studied. One explanation for the failure to reduce noise exposure can be that self-hygiene of the staff is often secondary while clinical activities to ensure the survival of patients predominate [60]. On the other hand, it can be countered that the noise sources listed are not all necessarily associated with acute medical or nursing interventions. Thus, it should be considered which aspects of noise management are necessary for, and relevant to the staff. In this context, Renz et al. [64] describe that at work, noise sources which are not important for one's own work and information intake are mainly perceived as disturbing (e.g. private conversations by colleagues).

### Strengths and limitations

To the best of our knowledge, this study provides a first insight into how unit-based noise management can reduce the subjective noise exposure and how this intervention affects other noise-related topics among staff in German ICUs over time. One of the main strengths is that we surveyed different types of healthcare professionals, such as physicians, nurses or therapists to capture different perspectives and ensure a comprehensive understanding of this issue. Another strength is that the intervention was unit-specific, recognizing that a one-size-fits-all approach is not appropriate. However, the study also has some limitations. One limitation is that we did not adjust for multiple testing in the significant tests on "noise-related topics" or the "individual noise sources". Thus, the significant results could be random. However, due to our exploratory approach, we did not want to be too restrictive. Another limitation is that we mainly considered complete cases in our analysis. With respect to noise-related strain, we used an additional statistical method (LMM) including all the observations. In addition, the study was monocentric and the response rate for all MPs ranged from 33.68% to 41.81%, which may reduce the representativeness (i.e. nonresponse bias [65]) and generalizability of our results.

## Conclusions

In summary, the present study aimed to explore the impact of unit-based noise management on staff's subjective noise exposure and other noise-related concerns in three ICUs. Results indicated that staff in the ICUs experienced substantial noise exposure. Nevertheless, the study failed to identify a significant reduction in the perceived noise-related strain. Staff were cognizant of the noise issue and considered the implementation of noise-management as important. Future interventions should aim to minimize noise from the most disturbing and relevant sources. Moreover, it is imperative to research aspects of adherence and their facilitators or barriers, which promote the sustained implementation of noise-reducing measures by staff. We therefore encourage researchers to take these aspects into consideration while designing future studies. Furthermore, we are currently planning a corresponding research project ourselves.

### Supplementary Information

**Additional file 1. **Table with relative frequencies of the items of other noise-related topics.

**Additional file 2. **Table with relative frequencies of perceived disturbance of the noise sources.

## Data Availability

The datasets generated and/or analyzed in the current study are not publicly accessible due to data protection agreements within the project and a non-reasonable effort in performing a legally robust anonymization. Upon justified request, disclosure of anonymized data can be discussed with the authors in individual cases. The prerequisite for this is clarification of compatibility with the data protection regulations (including the consent declarations) and questions of the expense of a legally robust anonymization.

## Abbreviations

CFA: Confirmatory factor analysis
CI: Confidence interval
ICC: Intraclass correlation coefficient
ICU(s): Intensive care unit(s)
LMM: Linear mixed model
MP(s): Measurement point(s)
