# Supplementary material for: Noise exposure among staff in intensive care units and the effects of unit-based noise management: a monocentric prospective longitudinal study
Source: BMC Nurs. 2023 Dec 6;22:460. doi: 10.1186/s12912-023-01611-3 (PMC10699060; doi:10.1186/s12912-023-01611-3)
Supplement: Supplementary file 1 — Additional file 1. Table with relative frequencies of the items of other noise-related topics. [file 12912_2023_1611_MOESM1_ESM.pdf]

Additional file 1: Relative frequencies of the items of other noise-related topics

	All observations (T0)					CC (T0)[a]					CC (T0)[b]				
Topic/Item	n	no	rather no	rather yes	yes	n	no	rather no	rather yes	yes	n	no	rather no	rather yes	yes
**Knowledge and awareness**															
The World Health Organization recommends that the noise level in the hospital should not exceed 35-40 dB(A) (unit for noise level). The example " room ventilator" = 35 dB(A) should give you an orientation. Do you think this guideline is implemented most of the time?	106	53.8	38.7	7.5	-	59	54.2	39.0	6.8	-	47	55.3	42.6	2.1	-
Do you experience the sounds on the ICU as too loud?	112	4.5	8.0	48.2	39.3	62	4.8	9.7	46.8	38.7	50	10.0	6.0	54.0	30.0
Do you think it would be possible to reduce noise levels on the ICU, by changing your own behavior?	112	4.5	27.7	44.6	23.2	62	1.6	25.8	45.2	27.4	50	6.0	26.0	42.0	26.0
**Thematization**															
Do you think it is very important to thematize noise on ICUs?	112	-	1.8	22.3	75.9	62	-	-	17.7	82.3	50	-	-	26.0	74.0
Do you talk with your colleagues about the ICU ambient noise?	112	8.0	27.7	21.4	42.9	62	6.5	30.6	14.5	48.4	50	10.0	30.0	24.0	36.0
Is the ambient noise on the ICU an issue in discussions with your superiors?	112	24.1	36.6	16.1	23.2	62	21.0	38.7	11.3	29.0	49	36.6	34.7	10.2	24.5
Do you talk with your family/friends about the noise on the ICU?	111	35.1	35.1	15.3	18.0	61	34.4	26.2	18.0	21.3	50	36.0	28.0	22.0	14.0
Are you addressed by patients or their dependents about the noise levels on the ICU?	112	15.2	40.2	27.7	17.0	61	16.4	39.3	26.2	18.0	50	18.0	36.0	30.0	16.0
**Subjective noise-sensitivity**															
Are you seeking increased for calm after a working shift on the ICU?	112	7.1	21.4	35.7	35.7	62	6.5	22.6	38.7	32.3	50	10.0	18.0	42.0	30.0
Do you feel being lesser in the mood for listening to music after working?	111	28.8	28.8	21.6	20.7	61	27.9	32.8	19.7	19.7	49	32.7	30.6	18.4	18.4
Compared to other people who don´t work on the ICU: Do you react more sensitive to sounds after having had a shift on the ICU?	108	17.6	24.1	32.4	25.9	56	21.4	25.0	26.8	26.8	47	21.3	25.5	27.7	25.5
**Attitude towards alternative alarm systems**															
Do you think, vibrating alarm signals (e.g. by a smart watch) could replace auditive ones (e.g. a call system)?	112	7.1	27.7	39.3	25.9	61	6.6	24.6	41.0	27.9	50	14.0	22.0	32.0	32.0
Do you think, visible alarm signals (e.g. by a smart watch) could replace auditive ones (e.g. a call	111	8.1	42.3	32.4	17.1	61	6.6	32.8	39.3	21.3	49	14.3	30.6	30.6	24.5

system)?

[a] Data for T0 refer to the staff who participated in the survey at T0 and T1; [b] data for T0 refer to the staff who participated in the survey at T0 and T2
