# Supplementary material for: Noise exposure among staff in intensive care units and the effects of unit-based noise management: a monocentric prospective longitudinal study
Source: BMC Nurs. 2023 Dec 6;22:460. doi: 10.1186/s12912-023-01611-3 (PMC10699060; doi:10.1186/s12912-023-01611-3)
Supplement: Supplementary file 2 — Additional file 2. Table with relative frequencies of perceived disturbance of the noise sources. [file 12912_2023_1611_MOESM2_ESM.pdf]

Additional file 2: Relative frequencies of the perceived disturbance of all noise sources

	All observations (T0)						CC (T0)[a]						CC (T0)[b]					
	n	don`t know	not at all disturbing	rather not disturbing	rather disturbing	very disturb ing	n	don`t know	not at all disturbing	rather not disturbing	rather disturbing	very disturb ing	n	don`t know	not at all disturbing	rather not disturbing	rather disturbing	very disturb ing
**Technical devices**																		
Mechanical ventilators	111	1.8	5.4	28.8	39.6	24.3	61	-	6.6	24.6	44.3	24.6	49	-	8.2	26.5	40.8	24.5
Surveillance monitors (alarms)	112	-	1.8	8.0	39.3	50.9	62	-	3.2	11.3	30.6	54.8	48	-	4.2	8.3	41.7	45.8
Dialysis machine	111	25.2	8.1	30.6	19.8	16.2	61	27.9	9.8	27.9	14.8	19.7	50	24.0	10.0	28.0	16.0	22.0
Perfusors	112	2.7	4.5	25.0	43.8	24.1	62	3.2	4.8	22.6	48.4	21.0	50	2.0	8.0	24.0	48.0	18.0
ECMO[c]	106	52.8	7.5	27.4	10.4	1.9	59	57.6	6.8	22.0	10.2	3.4	48	47.9	10.4	25.0	14.6	2.1
Suction pump	111	9.0	9.0	44.1	32.4	5.4	60	11.7	10.0	40.0	35.0	3.3	49	14.3	14.3	30.6	34.7	6.1
Visitor bell	112	4.5	7.1	33.9	33.0	21.4	62	3.2	8.1	32.3	32.3	24.2	50	2.0	12.0	30.0	30.0	26.0
Telephones	112	1.8	2.7	16.1	38.4	41.1	62	1.6	3.2	16.1	35.5	43.5	50	-	6.0	24.0	30.0	40.0
Beeper	110	9.1	12.7	35.5	27.3	15.5	59	6.8	13.6	42.4	22.0	15.3	48	4.2	22.9	31.2	25.0	16.7
Heated blanket	111	30.6	12.6	38.7	12.6	5.4	60	36.7	10.0	36.7	11.7	5.0	49	38.8	14.3	26.5	14.3	6.1
Compressed air	110	26.4	11.8	40.9	19.1	1.8	59	30.5	10.2	35.6	20.3	3.4	48	22.9	16.7	37.5	20.8	2.1
Thoracic drainage	109	22.9	21.1	45.0	10.1	0.9	58	25.9	24.1	37.9	10.3	1.7	45	15.6	31.1	40.0	11.1	2.2
Transport monitor / ventilator	110	7.3	7.3	48.2	22.7	14.5	60	8.3	10.0	46.7	18.3	16.7	49	4.1	14.3	46.9	22.4	12.2
**Clinical activities or actions**																		
Using the brake on the bed	111	12.6	9.0	18.9	25.2	34.2	61	14.8	14.8	8.2	24.6	37.7	50	18.0	12.0	14.0	18.0	38.0
Opening cartons or packages	110	-	11.8	34.5	25.5	28.2	58	-	15.5	25.9	19.0	39.7	48	-	16.7	25.0	20.8	37.5
Opening or closing doors/drawers	111	-	8.1	50.5	24.3	17.1	62	-	6.5	48.4	22.6	22.6	49	-	10.2	46.9	24.5	18.4
Visits	112	0.9	8.9	34.8	30.4	25.0	62	-	8.1	35.5	33.9	22.6	50	-	8.0	36.0	34.0	22.0
Private conversations from colleagues	112	3.6	2.7	26.8	36.6	30.4	62	3.2	-	22.6	40.3	33.9	50	4.0	4.0	32.0	32.0	28.0
Cleaning work	112	0.9	12.5	41.1	28.6	17.0	62	-	16.1	37.1	25.8	21.0	49	-	20.4	40.8	18.4	20.4
Shoes (e.g. squeaking)	112	2.7	17.9	41.1	28.6	9.8	61	3.3	16.4	41.0	24.6	14.8	49	-	20.4	34.7	28.6	16.3
Conversation of visitors	111	2.7	12.6	53.2	22.5	9.0	60	3.3	8.3	53.3	25.0	10.0	48	2.1	16.7	54.2	18.8	8.3

[a] Data for T0 refer to the staff who participated in the survey at T0 and T1; [b] data for T0 refer to the staff who participated in the survey at T0 and T2; [c] extracorporeal membrane oxygenation
